# Inflammatory cell infiltrates, hypoxia, vascularization, pentraxin 3 and osteoprotegerin in abdominal aortic aneurysms – A quantitative histological study

**DOI:** 10.1371/journal.pone.0224818

**Published:** 2019-11-08

**Authors:** Tereza Blassova, Zbynek Tonar, Petr Tomasek, Petr Hosek, Ivana Hollan, Vladislav Treska, Jiri Molacek

**Affiliations:** 1 Department of Histology and Embryology and Biomedical Centre, Faculty of Medicine in Pilsen, Charles University, Pilsen, Czech Republic; 2 Hospital for Rheumatic Diseases, Lillehammer, Norway; 3 Department of Medicine, Brigham and Women’s Hospital, Boston, Massachusetts, United States of America; 4 Department of Vascular Surgery, University Hospital in Pilsen, Faculty of Medicine in Pilsen, Charles University, Pilsen, Czech Republic; Stellenbosch University Faculty of Medicine and Health Sciences, SOUTH AFRICA

## Abstract

Information about the tissue characteristics of abdominal aortic aneurysms (AAAs), some of which may be reflected in the serum, can help to elucidate AAA pathogenesis and identify new AAA biomarkers. This information would be beneficial not only for diagnostics and follow-up but also for potential therapeutic intervention. Therefore, the aim of our study was to compare the expression of structural proteins, immune factors (T and B lymphocytes, macrophages, neutrophils and pentraxin 3 (PTX3)), osteoprotegerin (OPG), microvessels and hypoxic cells in AAA and nonaneurysmal aortic walls. We examined specimens collected during surgery for AAA repair (n = 39) and from the abdominal aortas of kidney donors without AAA (n = 8). Using histochemical and immunohistochemical methods, we quantified the areas positive for smooth muscle actin, desmin, elastin, collagen, OPG, CD3, CD20, MAC387, myeloperoxidase, PTX3, and hypoxia-inducible factor 1-alpha and the density of CD31-positive microvessels. AAA samples contained significantly less actin, desmin, elastin and OPG, more collagen, macrophages, neutrophils, T lymphocytes, B lymphocytes, hypoxic cells and PTX3, and a greater density of vasa vasorum (VV) than those in non-AAA samples. Hypoxia positively correlated with actin and negatively correlated with collagen. Microvascular density was related to inflammatory cell infiltrates, hypoxia, PTX3 expression and AAA diameter. The lower OPG expression in AAAs supports the notion of its protective role in AAA remodeling. AAA contained altered amounts of structural proteins, implying reduced vascular elasticity. PTX3 was upregulated in AAA and colocalized with inflammatory infiltrates. This evidence supports further evaluation of PTX3 as a candidate marker of AAA. The presence of aortic hypoxia, despite hypervascularization, suggests that hypoxia-induced neoangiogenesis may play a role in AAA pathogenesis. VV angiogenesis of the AAA wall increases its vulnerability.

## Introduction

Abdominal aortic aneurysms (AAAs) occur in 1–7% of the population over 50 years of age [1]. The pathomechanisms underlying the development of AAAs and AAA instability, which may induce AAA disruption, are still unclear. Therefore, the prevention and treatment of AAAs are insufficient. Furthermore, tools for monitoring AAAs and predicting their complications are limited.

Thus, it is important to identify the crucial structural changes and processes that lead to the development of AAAs and AAA instability. Some of these may be reflected in the serum, serve as biomarkers for diagnosing and monitoring AAAs, and predict their complications. Moreover, improved insights into the pathophysiological processes may help to identify novel therapeutic targets.

AAAs are characterized by decreased vascular elasticity. There are theories that inflammation and changes in microcirculation can contribute to the vascular remodeling of aneurysms [2–5]. Aortic inflammatory cells (T and B lymphocytes) and endothelial cells from invading neovessels express matrix metalloproteinases (MMP) and may substantially contribute to aneurysm instability [6]. Nonetheless, there have been inconsistent results regarding the vascularization of AAAs; while a study by Eberlová revealed lower microvascular density in AAAs, Rodella found a higher density of microvessels in the AAA aorta compared to the non-AAA aorta [2,4].

Among the factors that may be involved in the pathogenesis of AAAs are osteoprotegerin (OPG) and pentraxin 3 (PTX3). PTX3 is a molecule of the innate immune system that protects against infections, participates in the clearance of apoptotic cells, modulates inflammation and angiogenesis, and participates in extracellular matrix formation. PTX3 belongs to the same protein family as C-reactive protein (CRP). However, in contrast to CRP, it is produced locally in the inflamed tissue and in neutrophils [7]. There are indications that PTX3 may be superior to CRP as a biomarker of atherosclerotic cardiovascular diseases (CVD) (including acute coronary syndromes), possibly due to its ability to reflect vascular inflammation and due to the speed of its response [8–10]. Interestingly, the role of PTX3 in CVD may be protective, and PTX3 may represent a relevant therapeutic target [11,12]. Nevertheless, there is currently minimal knowledge about the role of PTX3 in AAAs.

OPG, a key regulator of bone remodeling, has also been implicated in the immune response and vascular diseases. OPG is secreted by osteoblasts, endothelial cells, human aortic vascular smooth muscle cells (VSMCs), dendritic cells, lymphocytes and plasma cells [13]. OPG inhibits vascular calcification by regulating the procalcific effects of receptor activator of nuclear factor kappa-B ligand in VSMCs [14,15]. The role of OPG in CVD has not yet been fully clarified. Clinical studies have shown that high OPG levels are related to the presence and progression of CVD, including AAAs [13,16,17]. However, animal models point to a protective role of OPG in CVD [18,19].

In order to improve insights into vessel wall alterations in AAAs, we compared the expression of structural proteins, osteoprotegerin, and pentraxin 3 and the presence of immune factors (T and B lymphocytes, neutrophils and macrophages), microvessels and hypoxic cells in AAA and non-aneurysmal aortic walls and to explore their relationships.

## Materials and methods

### Patients

In this study, we examined aortic tissue removed during open surgical repair of AAA from 39 patients, and corresponding aortic specimens from 8 individuals—cadaveric organ donors without aortic aneurysms. In the AAA group, the inclusion criteria were a diagnosis of AAA and open surgery at University Hospital in Pilsen. The exclusion criteria were malignancy in the anamnesis, infection or autoimmune disease, and renal and hepatic dysfunction. There were no inflammatory AAAs in our group of patients as well as no familial AAAs. Also all AAAs were non-ruptured. Control samples were from heart-beating donors after brain death diagnosed. The samples were taken together with other organs for transplantation according to the Transplantation Act valid in the Czech Republic. Thus, the samples did not suffer any warm ischemia. The aortas were flushed with a perfusion solution together with the organs, and immediately after explantation they were fixed in formalin solution. This study conforms to the principles outlined in the Declaration of Helsinki. The study was approved by the Ethical Committee of University Hospital and the Faculty of Medicine of Charles University in Pilsen on 12th August 2014, and all the AAA patients gave written informed consent.

### Histological analysis

The specimens were fixed in formalin and embedded in paraffin, and each was cut into 28 serial 4-μm thick histological sections. The sections were stained using a battery of 14 methods to assess of overall morphology and markers of main tissue components, immune factors, hypoxia, osteoprotegerin and microvessels (Table 1). General protocol for immunohistochemical methods can be find here: dx.doi.org/10.17504/protocols.io.7uxhnxn. S1 Table provides details regarding the antibodies and pretreatment used in the immunohistochemical methods. The immunohistochemical reactions were visualized with diaminobenzidine (DAB+, Liquid; DakoCytomation, Glostrup, Denmark). The immunohistochemical sections were counterstained with Gill’s haematoxylin.

**Table 1:** Histological staining methods used in the study.

Staining	Purpose and visualization of aortic wall components
Hematoxylin-eosin [20]	Overall morphology of the aortic wall
Verhoeff’s hematoxylin and green trichrome [21]	Overall morphology, differentiating connective tissue, smooth muscle
Picrosirius red (Direct Red 80, Sigma Aldrich, Munich, Germany) [22]	Type I and type III collagen when observed under circularly polarized light
Orcein (Tanzer’s orcein, Bowley Biochemical Inc., Danvers, MA, USA)	Elastic membranes, elastic fibres
Immunohistochemical detection of alpha-smooth muscle actin	Contractile phenotype of vascular smooth muscle cells
Immunohistochemical detection of desmin	Contractile phenotype of vascular smooth muscle cells
Immunohistochemical detection of MAC387	Macrophages infiltrating the aortic wall
Immunohistochemical detection of myeloperoxidase	Neutrophilic granulocytes infiltrating the aortic wall
Immunohistochemical detection of CD3	T-lymphocytes
Immunohistochemical detection of CD20	B-lymphocytes
Immunohistochemical detection of CD31	Endothelium of vasa vasorum
Immunohistochemical detection of HIF 1-alpha	Hypoxia-inducible factor 1-alpha, a marker of tissue hypoxia
Immunohistochemical detection of Pentraxin-3	Pentraxin 3, a protein produced in response to inflammatory signals
Immunohistochemical detection of Osteoprotegerin	Osteoprotegerin, a calcium controlling protein

For each staining method, 4 micrographs were randomly collected in a systematic and uniform manner; resulting in total 56 micrographs for each sample. We used 20× and 40× objectives mounted on an Olympus BX51 microscope to take the photomicrographs (see S2 Table). The unbiased sampling of the micrographs of the sections was performed as described in our previous studies on the aorta [23,24]. Briefly, starting in a randomly selected part of a histological section, the *(x*, *y)* distances between 4 micrographs uniformly covered the entire circumference of the tunica media, including the image fields bordering the adventitia or the lumen. This resulted in a fair sampling of the image fields, in which all of the components and structures were selected with a probability proportional to their areas on the histological slide. To quantify collagen by microscopy, we used a circular polarizing filter (Hama, Monheim, Germany) crossed with a quarter wave λ/4 filter below the analyser filter (U-GAN, Olympus, Tokyo, Japan) mounted on an Olympus CX41 microscope (Olympus, Tokyo, Japan). The advantages of this method are described elsewhere [22].

Histological quantification was performed as described previously [2,23–28]. Briefly, a stereological point grid (the PointGrid module of Ellipse software (ViDiTo, Košice, Slovakia), cf. [29]) was loaded and randomly superposed on the micrographs to quantify the area of actin, desmin, elastin, collagen staining and the immunopositivity of macrophages, neutrophils, T lymphocytes, B lymphocytes, hypoxic cells, PTX3, and OPG. The number of points that intersected the structure of interest was counted. The point grid method allowed for individual corrections of the reference space (i.e., tunica media or adventitia) for any possible artefacts, microcracks or the presence of the lumen vs. the vessel wall. Whenever possible, the entire wall was used as a meaningful reference space [30]. The area for each parameter was calculated for each protein by dividing the number of grid lines intersecting the structure of interest by the number of grid lines intersecting the reference space of the vascular wall, and the result was multiplied by 100. The vascularization of the wall was quantified using an unbiased counting frame positioned on the micrographs. The number of CD31-positive vasa vasorum (VV) profiles was divided by the sum of the areas of the counting frame and expressed as a two-dimensional density of microvessels (Q_A_, quantity per area).

### Statistics

In some cases, the surgeon took two samples from one patient. We processed both samples and used the average results for the statistical analysis. We used the chi-square test to assess differences in proportions among the groups. We compared continuous nonnormally distributed variables by the Mann-Whitney-Wilcoxon test. Correlations were assessed using the Spearman correlation coefficient and Kendall's tau coefficient. All tests were two-sided and performed with the Statistica Base 11 package (StatSoft, Inc., Tulsa, OK, USA). The level of statistical significance was set at 0.05. False discovery rate (FDR) was controlled using the Benjamini-Hochberg procedure carried out upon the results of all significance test performed within the study. At the baseline significance level of 0.05, the estimated FDR is 20%, indicating 80% of the presented significant results to be true positives. A conservative overall FDR of 5% would require the individual significance level of 0.005.

The complete primary morphometric data from our samples (see S3 Table) have been made publicly available for further analyses.

## Results

Characteristics of the examined individuals are presented in Table 2. The AAA group showed a trend toward a higher proportion of males and a significantly higher mean age.

**Table 2:** Data of patients.

	AAA patient (n = 39)	Control group (n = 8)	p value
Male n (%)	32 (82%)	4 (50%)	0.057
Age (years)	70 (6.7)	51 (10.4)	0.00024
Average size of aneurysm (mm)	55 (11.8)	Not applicable	
Diabetes mellitus	8 (18%)	1 (13%)	NS
Hypertension	27 (61%)	3 (38%)	NS
Ischemic heart disease	18 (41%)	2 (25%)	NS
Peripheral artery disease	12 (27%)	4 (50%)	NS
Smoker—current	22 (56%)	3 (38%)	NS
Smoker—former	9 (23%)	2 (25%)	NS
Non-smoker	8 (21%)	3 (38%)	NS

Values are the median (SD) or frequency (relative frequency). NS–not significant.

**Fig 1:**
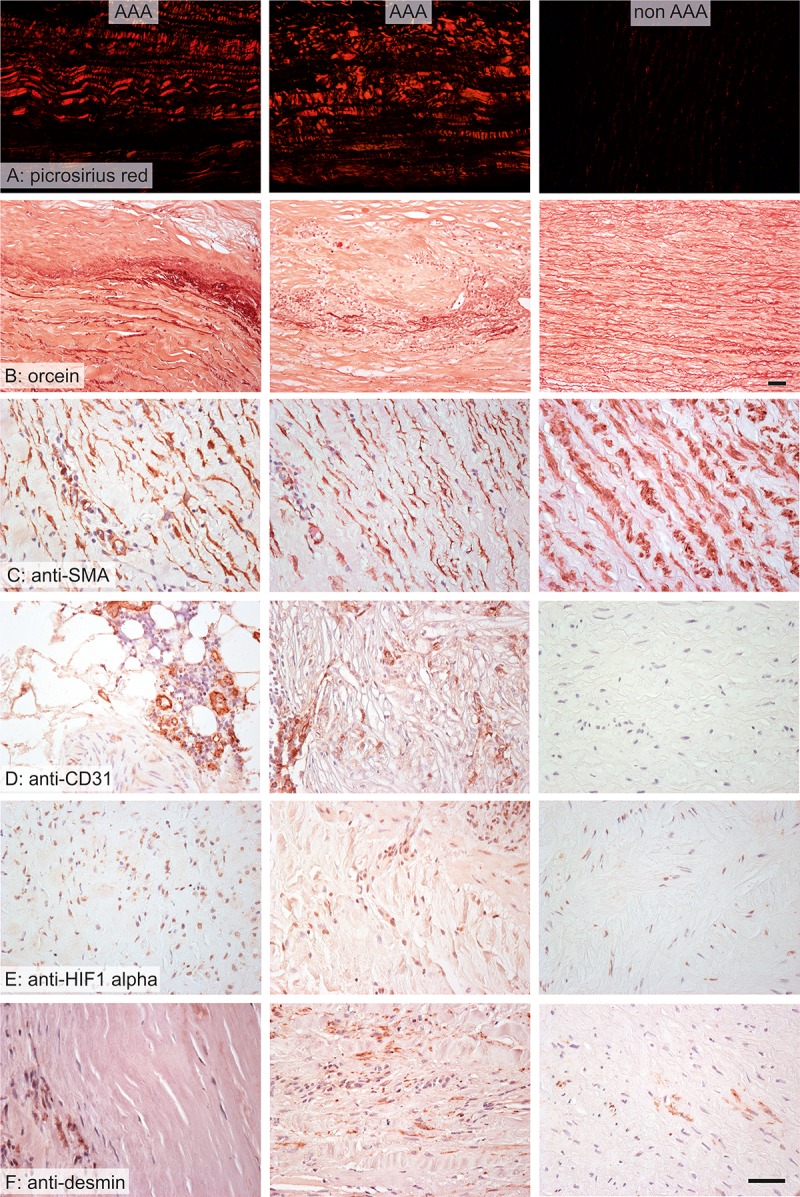
Wall composition in AAA (left and middle columns) and non-AAA samples (right column). In the AAA samples, the collagen was more abundant (A), the elastin was partially or mostly destroyed (B), the wall contained more vasa vasorum (D), expressed more hypoxia markers (E) and less contractile phenotype of vascular smooth muscle (C, F). Stained with picrosirius red (A), orcein (B), and immunohistochemistry with anti-smooth muscle actin antibody (C) for visualization of the contractile phenotype of vascular smooth muscle cells, anti-CD31 (D) for visualization of endothelium, anti-HIF 1-alpha (E) for visualization of tissue hypoxia and anti-desmin (F) for visualization of the contractile phenotype of vascular smooth muscle cells; nuclei were stained with Gill’s hematoxylin; scale bar 50 μm.

**Fig 2:**
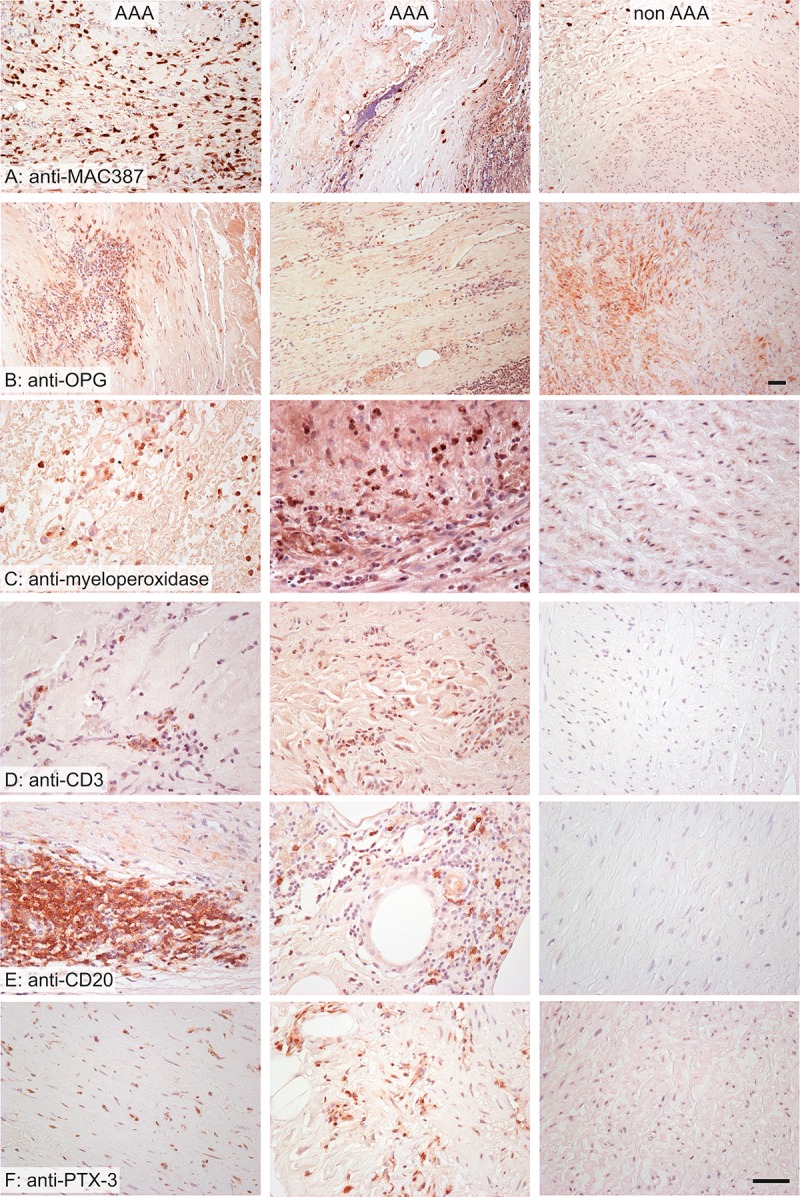
Wall composition in AAA (left and middle column) and non-AAA samples (right column). The AAA samples were more infiltrated by macrophages (A), neutrophils (B), T lymphocytes (D) and B lymphocytes (E). The distribution of osteoprotegerin (OPG) was more diffuse in non-AAA (C). The expression of pentraxin 3 was greater in AAA samples (F). Stained immunohistochemistry with anti-MAC387 antibody (A) for visualization of macrophages, anti-osteoprotegerin (B), anti-myeloperoxidase (C) for visualization of neutrophilic granulocytes, anti-CD3 (D) for visualization of T lymphocytes, anti-CD20 (E) for visualization of B lymphocytes, anti-pentraxin 3 (F); nuclei were stained with Gill’s hematoxylin; scale bar 50 μm.

Comparisons of AAA and non-AAA samples are summarized in Table 3. AAA samples contained significantly less actin, desmin, elastin (Fig 1) and osteoprotegerin, more collagen, macrophages, neutrophils, T lymphocytes, B lymphocytes, hypoxic cells and PTX3, and a greater density of VV (Fig 2) than did non-AAA samples. Microvascular density was related to inflammatory cell infiltrates, PTX3 expression and hypoxia.

**Table 3:** Testing the differences between the AAA vs. the non-AAA samples of aortic wall.

	AAA			Non AAA			
Parameter	Median	Lower Quartile	Upper Quartile	Median	Lower Quartile	Upper Quartile	p-value
*A*_*A*_*(actin*,*int+media)*	2.1	1.8	3.1	15.3	11.1	17.0	0.000013
*A*_*A*_*(desmin*,*int+media)*	0.3	0.1	0.4	0.6	0.4	0.7	0.025
*A*_*A*_*(elastin*,*int+media)*	0.8	0.2	1.4	3.9	3.1	5.2	0.00015
*A*_*A*_*(collagen*,*int+media)*	9.0	3.2	13.4	1.2	0.7	5.1	0.0034
*A*_*A*_*(MAC387*, *wall)*	1.2	0.9	1.8	0.7	0.5	1.0	0.039
*A*_*A*_*(myeloperoxidase*, *wall)*	2.5	2.0	3.0	1.2	1.0	2.6	0.046
*A*_*A*_*(CD3*, *wall)*	0.4	0.3	0.5	0.2	0.1	0.3	0.009
*A*_*A*_*(CD20*, *wall)*	0.7	0.4	1.0	0	0	0.2	0.0001
*A*_*A*_*(HIF 1-alpha*, *wall)*	0.9	0.6	1.0	0.3	0.2	0.4	0.001
*A*_*A*_*(osteoprotegerin*, *wall)*	0.6	0.5	0.9	1.4	1.3	1.8	0.001
*A*_*A*_*(pentraxin-3*, *wall)*	0.7	0.5	0.9	0.4	0.3	0.5	0.007
*Q*_*A*_*(CD31-positive microvessels*,*wall) (mm*^*-2*^*)*	74.4	52.4	98.1	37.2	24.5	38.9	0.0006

A_A_*(component*, *space)*: Area fraction of the respective components within their reference spaces (%); Q_A_: number of microvessel profiles per section area; *int+media*: data pooled from the intima and media; *wall*: data pooled the wall (i.e., from intima, media and adventitia). The abbreviations of all the parameters are explained in S2 Table.

**Fig 3:**
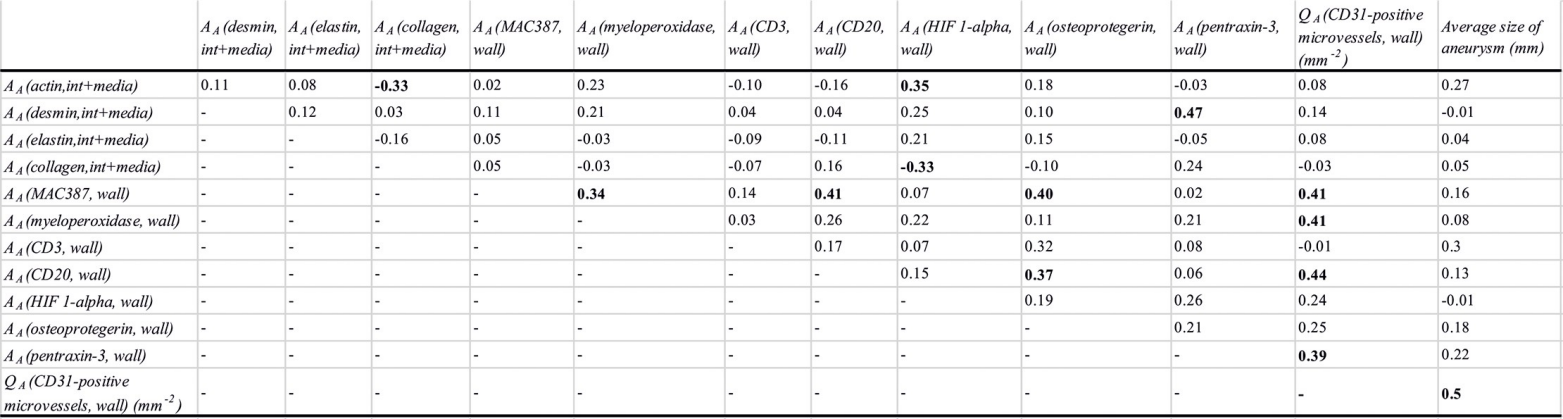
Correlations of the examined histological parameters, in AAA samples. All correlations significant at p<0.05 are highlighted in boldface. A_A_*(component*, *space)*: Area fraction of the respective components within their reference spaces; Q_A_: number of microvessel profiles per section area; *int+media*: data pooled from the intima and media; *wall*: data pooled from the wall (i.e., from intima, media and adventitia). Abbreviations of all the examined parameters are explained in S2 Table.

**Fig 4:**
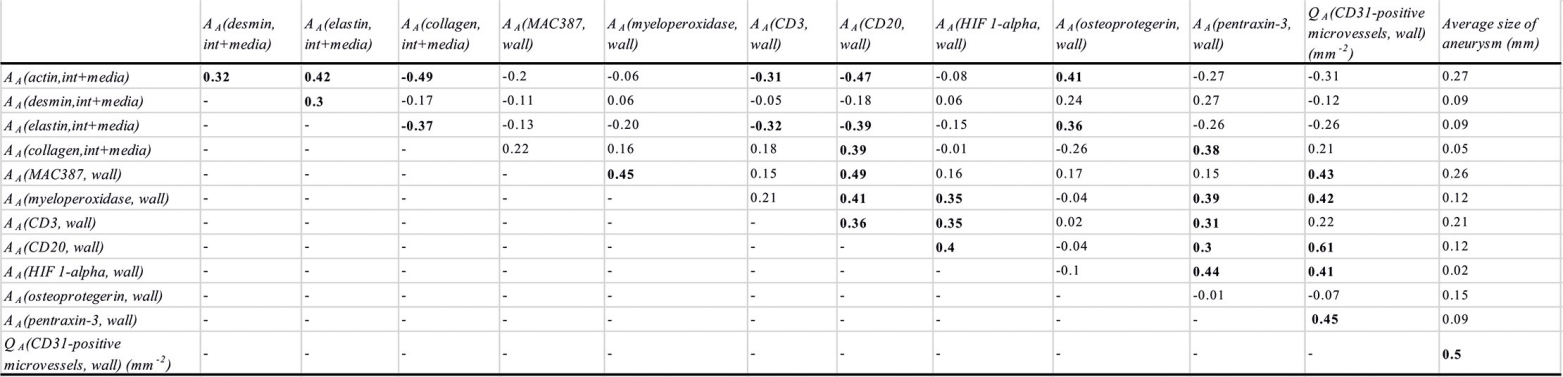
Correlations of the examined histological parameters, in all samples. All correlations significant at p<0.05 are highlighted in boldface. A_A_*(component*, *space)*: Area fraction of the respective components within their reference spaces; Q_A_: number of microvessel profiles per section area; *int+media*: data pooled from the intima and media; *wall*: data pooled from the wall (i.e., from intima, media and adventitia). The abbreviations of all the parameters are explained in S2 Table.

The correlations among the quantitative parameters among AAA patients are presented in Fig 3 and those among all examined individuals are presented in Fig 4.

Microvascular density was moderately positively correlated with hypoxia, neutrophils, macrophages, B lymphocytes and the clinically measured size of aneurysms in all samples and with neutrophils, macrophages, B lymphocytes and the aneurysm size in AAA samples.

OPG was positively moderately correlated with actin and elastin in all samples. OPG was positively moderately correlated with macrophages and B lymphocytes in the wall of AAA.

Markers of hypoxic cells were positively moderately correlated with neutrophils, T and B lymphocytes and patient age in all samples, while in AAA samples, the hypoxic cells were moderately positively correlated with actin and negatively correlated with collagen.

**Fig 5:**
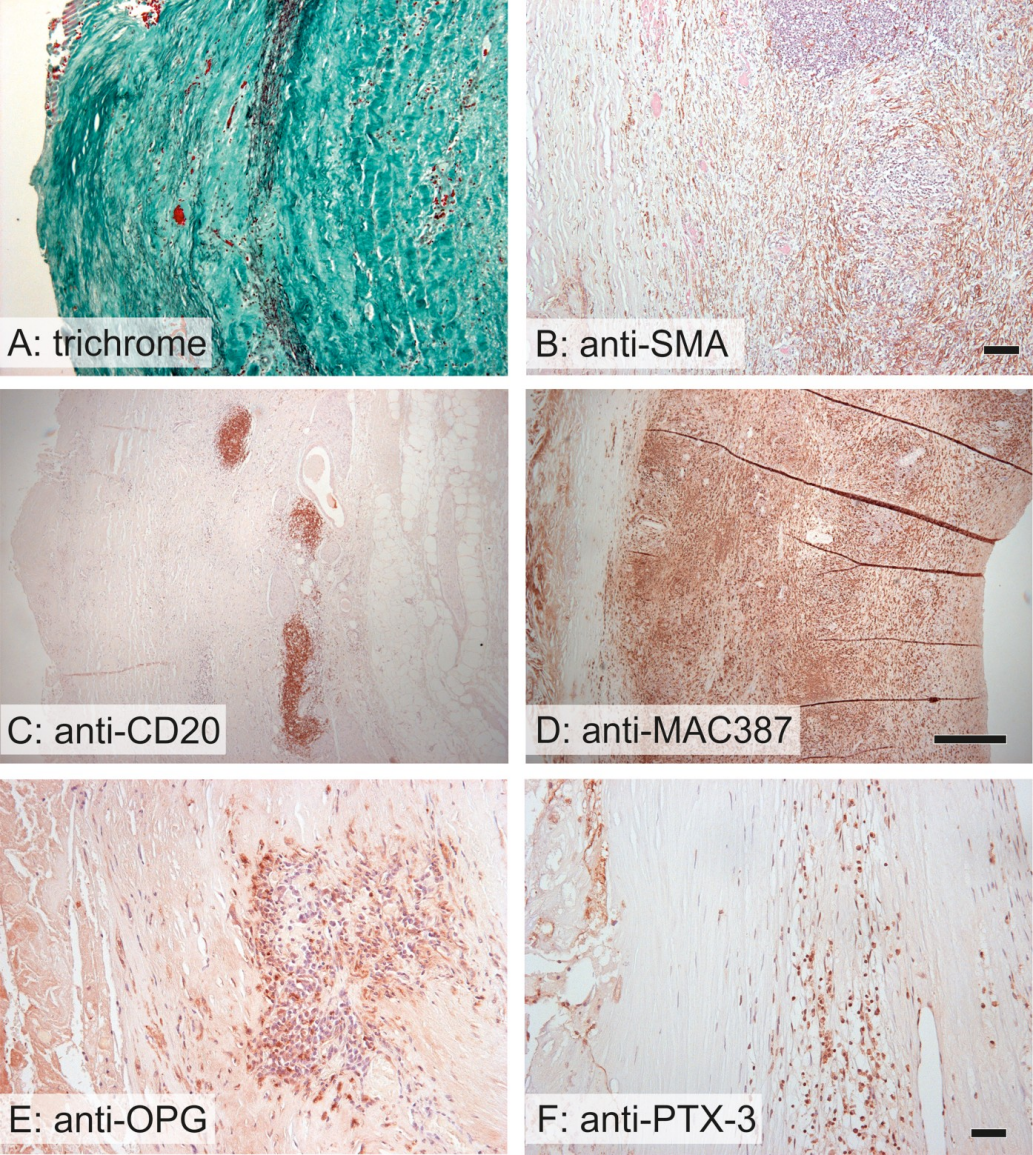
Qualitative findings in AAA samples. A—The basic pattern of the aortic wall was destroyed by newly formed vessels surrounded by inflammatory infiltrates. The elastin fibers (black) were compressed and destroyed. B–The inflammatory cells penetrated among the smooth muscle cells (brown) into the tunica media. C–Lymphocytes (brown) occurred mostly in aggregates resembling lymphoid follicles. D—Rarely, the macrophages (brown) penetrated diffusely the whole wall of the AAA. E–The expression of osteoprotegerin (brown) occurred mostly in the areas infiltrated by leukocytes. F–Similarly, the positivity of pentraxin 3 (brown) also occurred in areas infiltrated by leukocytes. Stained with Verhoeff’s hematoxylin and green trichrome (A) and immunohistochemistry with an antibody against smooth muscle actin (B) for visualization of the contractile phenotype of vascular smooth muscle cells, anti-CD20 (C) for visualization of B lymphocytes, anti-MAC387 (D) for visualization of macrophages, anti-osteoprotegerin (anti-OPG) (E) and anti-pentraxin 3 (anti-PTX3) (F); nuclei were stained with Gill’s hematoxylin; scale bar 100 μm (A, B), 500 μm (C, D) and 50 μm (E, F).

Men had fewer hypoxic cells than women (p = 0.001) in the AAA samples. Age had only a weak effect on the vascular density (r = 0.3; p = 0.032) in AAA samples. There was no influence of smoking on the evaluated histological parameters. The qualitative histological findings are shown as a composite in Fig 5. Our primary data from histological quantitative analysis are shown in S3 Table.

## Discussion

The aim of this study was to identify markers to recognize the presence and vulnerability of AAA because screening is not performed using Ultrasonography or Computer Tomography in most countries. Our AAA samples contained a higher microvessel density than non-AAA samples, which is in accordance with the findings of Rodella *et al*. [4]. As in the study by Eberlová *et al*., the microvessel density was correlated with the AAA diameter and the inflammatory infiltrates [2]. The number of microvessel profiles correlated positively with hypoxia and immune factors (B lymphocytes, macrophages, neutrophils and PTX3). The number of microvessel profiles correlated positively with hypoxia and immune factors (B lymphocytes, macrophages, neutrophils and PTX3). This correlation might be due to the stimulating effects of inflammatory and hypoxic states on angiogenesis [31]. In human AAA, the adventitial VV has been observed to be stenotic in both small and large AAA, with the sac tissue in these AAA being ischemic and hypoxic. Hypoperfusion of the vascular wall vessel could have critical effects on the development of infrarenal AAA [5]. This hypothesis was verified in a rodent model of AAA when VV blood flow was blocked through the tight ligation of the aorta over the catheter [32]. However, the reason for ischemia formation is still unclear. There were indications that the reduced oxygenation of the aortic wall might be due to reduced oxygen diffusion from the lumen due to intraluminal thrombus (ILT) [33]. Notably, more recent analyses indicate that changes in adventitial VV occurred, irrespective of the presence of ILT. Adventitial VV may play an independent role in the perfusion and oxygenation of the aortic wall, with VV stenosis contributing to the ischemia of the aortic wall itself [34]. Furthermore, increased aortic stiffness and hypertension may narrow the lumen of VV. Greater vascular density in AAA was found in older AAA patients. This result is in accordance with the findings [35] that showed an increased VV diameter and area within thoracic aorta in aged subjects. The AAA samples contained a greater number of hypoxic cells than did the non-AAA samples. The number of hypoxic cells correlated positively with immune factors (T and B lymphocytes and neutrophils). There are indications from other research that HIF 1-alpha is pivotal for AAA progression toward rupture [36]. Several factors related to aneurysm susceptibility (including angiotensin II and nicotine) cause the upregulation of MMP-2 and MMP-9 through aberrantly induced HIF 1-alpha and promote aneurysmal progression [37]. We demonstrated that men had fewer hypoxic cells than women in AAA patients. In a study of samples from patients with pulmonary arterial hypertension, the expression of HIF 1-alpha was higher in female than in male pulmonary artery smooth muscle cells [38]. There are differences in HIF 1-alpha signaling between female and male vascular smooth muscle cells [38]. This difference should be analyzed futher, since these findings could be useful for understanding the pathomechanisms of AAA in different genders.

We demonstrated a statistically significantly higher expression of PTX3 in AAA than in non-AAA patients. The PTX3 correlated positively with collagen, hypoxia and immune cells (T and B lymphocytes and neutrophils). These results corresponded with the findings from studies in which the parameters from AAA were compared to samples of ascending aorta without AAA [39,40]. The findings from these studies were not compared with other structural components of AAA tissue. These authors also analyzed PTX3 serum levels. There was no difference between the serum levels of AAA and non-AAA patients. PTX3 expression negatively correlated with the maximum diameter of the aneurysm [39]. In a recent study, PTX3 serum levels in AAA patients were higher than those in non-AAA patients. Serum levels were AAA diameter-independent [41]. The PTX3 expression in our tissue samples also did not correlate with aneurysm diameter. Peak levels of PTX3 in patients with acute aortic dissection were associated with the amount of transient pleural fluid accumulation, which may be associated with inflammatory vascular permeability [42]. Based on our results, we hypothesized that PTX3 might play a role in the pathogenesis of AAA. However, the histological analysis reflected the outcome of the AAA remodeling. Therefore, more evidence is necessary.

The structure of PTX3 is similar to that of CRP, which is from the same pentraxin superfamily. CRP was also suggested as a biomarker of AAA [43–46]. In contrast to PTX3, which is expressed in circulating neutrophils as well as a variety of cells in inflamed tissue (including endothelial cells, fibroblasts, VSMCs and inflammatory cells) [40,47,48]. CRP is synthesized by hepatocytes upon stimulation by systemic proinflammatory cytokines. Therefore, PTX3 is a more accurate marker of the actual inflammatory state and mirrors local inflammation that does not necessarily lead to an increase in systemic cytokines (e.g., in vascular inflammation, even without systemic inflammation) [49]. PTX3 is also less influenced by total cholesterol, high-density lipoprotein, hemoglobin, smoking, obesity or gender [49,50]. Our study provides evidence of the distribution of PTX3, OPG and hypoxic cells within AAAs, but further analyses relating histological data to biochemical markers are needed.

The AAA samples contained less elastin and more macrophages, neutrophils, T and B lymphocytes and a greater density of VV. These data support the finding of the increasing gene expression of MMP-9, which degrades elastin and plays a role in the proliferation and migration of VSMCs, and intercellular adhesion molecule-1, which promotes leukocyte adhesion to and migration through endothelial cells [6,51].

The AAA samples contained a smaller amount of OPG. These findings from our quantitative analyses support a potential preventive effect of OPG [19].

A limitation of the present study is that the results were based on AAA samples harvested during an open surgical repair of AAA sac. However, many AAA patients undergo endovascular aortic repair [52], where no morphological tissue samples can be removed for research purposes. Moreover, also harvesting of control samples of healthy aorta from organ donors has limitations as the interest of the waiting organ recipients is prioritized over harvesting of samples for research purposes. In addition, bias may have been introduced since the morphometry of our samples was based on 1–2 histological sections per each quantitative parameter. Also, our quantification used two-dimensional routine sections only, whereas some structures (e.g. the vasa vasorum) are three-dimensional and would require more sophisticated quantification techniques, e.g. confocal microscopy or X-ray microtomography. Moreover, also the present study relies on histological analysis, but its conclusions should be further verified using established quantitative techniques (Enzyme-Linked Immunosorbent Assay or western blots) to demonstrate the differences in protein expression more convincingly.

## Conclusion

AAA tissue samples contained significantly less actin, desmin, elastin and osteoprotegerin, more collagen, macrophages, neutrophils, T lymphocytes, B lymphocytes, hypoxic cells and PTX3, and a greater density of VV than did non-AAA samples. PTX3 and hypoxia correlated with each other and with T and B lymphocytes and neutrophils. Microvascular density was related to inflammatory cell infiltrates, PTX3 expression, hypoxia and average size of aneurysms. AAA contained altered amounts of structural proteins, implying reduced vascular elasticity. This remodeling of the AAA wall occurred under significant tissue hypoxia, despite a greater density of microvessels than of non-aneurysmal aorta. PTX3 was upregulated in AAA, and its colocalization with the inflammatory infiltrates supports the theory of a potential role for PTX3 as a marker of vascular inflammation. The presence of aortic hypoxia, despite hypervascularization, suggests hypoxia-induced neoangiogenesis that may play a role in AAA pathogenesis. VV angiogenesis of the AAA wall increases its vulnerability.

## Supporting information

S1 Table Primary antibodies used for immunohistochemistry. (DOC)

S2 Table Quantitative parameters used for morphometry of the aortic wall. (DOC)

S3 Table Primary data from analysis. (XLS)
