# Supplementary material for: Inflammatory cell infiltrates, hypoxia, vascularization, pentraxin 3 and osteoprotegerin in abdominal aortic aneurysms – A quantitative histological study
Source: PLoS One. 2019 Nov 8;14(11):e0224818. doi: 10.1371/journal.pone.0224818 (PMC6839860; doi:10.1371/journal.pone.0224818)
Supplement: S1 Table — (DOC) [file pone.0224818.s001.doc]

| **Antibody** | **Imunogen** | **Catalog code** | **Manufacturer** | **Dilution factor** | **Pretreatment** | **Positive control** | **Ref.** |
| --- | --- | --- | --- | --- | --- | --- | --- |
| Monoclonal Mouse Anti‑Human Smooth Muscle Actin, Clone 1A4 *(smooth muscle phenotype marker)* | N-terminal synthetic decapeptide of α-smooth muscle actin | M085101-2 | DakoCytomation (Glostrup, Denmark) | 1:500 | Heat-induced epitope retrieval (HIER) in Epitope Retrieval Solution pH 9 (Novocastra Leica, Leica Biosystems GmbH, Nussloch, Germany); 20 minutes | Human colon | [1] |
| Monoclonal Mouse Anti‑Human Desmin, Clone D33 *(smooth muscle contractile phenotype marker)* | Desmin purified from human muscle | M076001-2 | DakoCytomation | 1:300 | HIER in Epitope Retrieval Solution pH 9 (Novocastra Leica); 20 minutes | Human colon | [2] |
| Polyclonal Rabbit Anti‑Human Myeloperoxidase *(marker of neutrophlic granulocytes)* | Myeloperoxidase isolated from human polymorphonuclear leucocytes | A039829-2 | DakoCytomation | 1:300 | HIER in Epitope Retrieval Solution pH 6 (Novocastra Leica); 20 minutes | Human spleen | [3] |
| Monoclonal Mouse Anti‑Human Macrophages/Monocytes Antibody, clone MAC387 *(marker of macrophages)* | Human monocytes | MA1-80446 | ThermoFischer Scientific (Rockford, IL, USA) | 1:200 | HIER in Epitope Retrieval Solution pH 9 (Novocastra Leica); 20 minutes | Human spleen | [3] |
| Monoclonal Rabbit Anti‑Human CD3e Antibody, clone SP7 *(marker of T-lymphocytes)* | Synthetic peptide KAKAKPVTRGAGA, corresponding to amino acids 156-168 of Human CD3 epsilon chain | MA1-90582 | ThermoFischer Scientific | 1:400 | HIER in Epitope Retrieval Solution pH 6 (Novocastra Leica); 10 minutes | Human thymus | [4] |
| Monoclonal Mouse Anti‑Human CD20 Antibody, clone L26 *(marker of B-lymphocytes)* | Human tonsil B cells | MA5-13141 | ThermoFischer Scientific | 1:400 | HIER in Epitope Retrieval Solution pH 6 (Novocastra Leica); 20 minutes | Human tonsil | [5] |
| Monoclonal Monoclonal Anti‑CD31 antibody Clone J70A *(endothelial marker)* | Cell membrane preparation from the spleen of a patient with hairy cell leukemia | M082301-2 | DakoCytomation | 1:100 | Proteinase K (DakoCytomation) | Human kidney | [3] |
| Polyclonal Rabbit Anti‑Human Osteoprotegerin Antibody *(marker of OPG)* | Synthetic peptide conjugated to KLH  derived from within residues 200 -  300 Osteoprotegerin | PA5-19841 | ThermoFischer Scientific | 1:500 | HIER in Epitope Retrieval Solution pH 9 (Novocastra Leica); 20 minutes | Human kidney | [6] |
| Monoclonal Mouse Anti‑Human HIF1A Antibody, clone H1alpha76 *(marker of tissue hypoxia)* | Fusion protein containing residues 432-528 of human HIF-1alpha | MA1-16504 | ThermoFischer Scientific | 1:100 | HIER in Epitope Retrieval Solution pH6 (Novocastra Leica); 20 minutes | Human colon | [7] |
| Polyclonal Rabbit Anti‑Human Pentraxin3 Antibody *(marker of PTX3)* | Recombinant protein corresponding to Human PTX3 | PA5-64410 | ThermoFischer Scientific | 1:500 | HIER in Epitope Retrieval Solution pH 9 (Novocastra Leica); 20 minutes | Human umbilical cord | [8] |

As a negative control, each immunohistochemical protocol was performed with the primary antibody omitted.

**References**

1. Buzgo M, Plencner M, Rampichova M, Litvinec A, Prosecka E, Staffa A, et al. Poly-ε-Caprolactone and Polyvinyl Alcohol Electrospun Wound Dressings: Adhesion Properties and Wound Management of Skin Defects in Rabbits. Regen Med. 2019;14: 423–445. doi:10.2217/rme-2018-0072
2. Tonar Z, Kubíková T, Prior C, Demjén E, Liška V, Králíčková M, et al. Segmental and Age Differences in the Elastin Network, Collagen, and Smooth Muscle Phenotype in the Tunica Media of the Porcine Aorta. Ann Anat Anat Anz Off Organ Anat Ges. 2015;201: 79–90. doi:10.1016/j.aanat.2015.05.005
3. Eberlová L, Tonar Z, Witter K, Křížková V, Nedorost L, Korabečná M, et al. Asymptomatic Abdominal Aortic Aneurysms Show Histological Signs of Progression: a Quantitative Histochemical Analysis. Pathobiol J Immunopathol Mol Cell Biol. 2013;80: 11–23. doi:10.1159/000339304
4. Patyka M, Malamud D, Weissman D, Abrams WR, Kurago Z. Periluminal Distribution of HIV-Binding Target Cells and Gp340 in the Oral, Cervical and Sigmoid/Rectal Mucosae: A Mapping Study. PLoS ONE. 2015;10: e0132942. doi:10.1371/journal.pone.0132942
5. Tekin B, Kempf W, Seckin D, Ergun T, Yucelten D, Demirkesen C. Interstitial Mycosis Fungoides With Lichen Sclerosus-Like Clinical and Histopathological Features. Am J Dermatopathol. 2016;38: 138–143. doi:10.1097/DAD.0000000000000406
6. Zhang R, Wan J, Wang H. Mechanical Strain Triggers Differentiation of Dental Mesenchymal Stem Cells by Activating Osteogenesis-Specific Biomarkers Expression. Am J Transl Res. 2019;11: 233–244.
7. Schreurs LMA, Smit JK, Pavlov K, Pultrum BB, Pruim J, Groen H, et al. Prognostic Impact of Clinicopathological Features and Expression of Biomarkers Related to (18)F-FDG Uptake in Esophageal Cancer. Ann Surg Oncol. 2014;21: 3751–3757. doi:10.1245/s10434-014-3848-6
8. Ke H-H, Hueng D-Y, Tsai W-C. Low Expression of Pentraxin 3 and Nuclear Factor-Like 2 Implying a Relatively Longer Overall Survival Time in Gliomas. Chinese Journal of Physiology. 2019;62: 35. doi:10.4103/CJP.CJP_3_19
