# Supplementary material for: Inflammatory cell infiltrates, hypoxia, vascularization, pentraxin 3 and osteoprotegerin in abdominal aortic aneurysms – A quantitative histological study
Source: PLoS One. 2019 Nov 8;14(11):e0224818. doi: 10.1371/journal.pone.0224818 (PMC6839860; doi:10.1371/journal.pone.0224818)
Supplement: S2 Table — (DOC) [file pone.0224818.s002.doc]

Quantitative parameters used for morphometry of the aortic wall.

| **Quantitative parameter abbreviation and units** | **Definition, reference area, and objective used for quantification** |
| --- | --- |
| *A_A_(actin, int+media)* (-) | The area fraction of the alpha-smooth muscle actin-positive cells within the intima and media reference area; 40×. |
| *A_A_(desmin, int+media)* (-) | The area fraction of the desmin-positive cells within the intima and media reference area; 40×. |
| *A_A_(elastin, int+media)* (-) | The area fraction of the elastin within the intima and media reference area; 20×. |
| *A_A_(collagen, int+media)* (-) | The area fraction of the collagen within the whole wall reference area; 20×. |
| *A_A_(MAC387, wall)* (-) | The area fraction of the MAC387-positive macrophages within the whole wall reference area; 20×. |
| *A_A_(myeloperoxidase, wall)* (-) | The area fraction of the myeloperoxidase-positive neutrophilic granulocytes within the whole wall reference area; 40×. |
| *A_A_(CD3, wall)* (-) | The area fraction of the CD3-positive T-lymphocytes within the whole wall reference area; 40×. |
| *A_A_(CD20, wall)* (-) | The area fraction of the CD20-positive B-lymphocytes within the whole wall reference area; 40×. |
| *A_A_(HIF 1-alpha, wall)* (-) | The area fraction of the HIF 1‑alpha-positive hypoxic cells within the whole wall reference area; 40×. |
| *A_A_(pentraxin-3, wall)* (-) | The area fraction of the pentraxin 3-positive cells within the whole wall reference area; 40×. |
| *A_A_(osteoprotegerin, wall)* (-) | The area fraction of the osteoprotegerin-positive cells within the whole wall reference area; 20×. |
| *Q_A_(CD31-positive microvessels,wall)* (mm^-2^) | Density of all CD31-positive vasa vasorum profiles per area unit of the whole section profile of the aortic wall; 40×. |
